# ECG and navigator-free 4D whole-heart coronary MRA

**DOI:** 10.1186/1532-429X-17-S1-O50

**Published:** 2015-02-03

**Authors:** Jianing Pang, Behzad Sharif, Zhaoyang Fan, Xiaoming Bi, Reza Arsanjani, Daniel S Berman, Debiao Li

**Affiliations:** 1 Biomedical Imaging Research Institute, Cedars-Sinai Medical Center, Los Angeles, CA, USA; 2 MR R&D, Siemens Healthcare, Los Angeles, CA, USA

## Background

Cardiac and respiratory motion artifacts are major challenges to whole-heart coronary MRA. The conventional motion suppression strategies often involve prospective gating based on motion surrogates, e.g. ECG and navigator, which complicates scan setup and prolongs scan time significantly. To address these limitations, an ECG and navigator-free 4D whole-heart coronary MRA technique was recently proposed, providing both cardiac function and coronary artery assessment from a single measurement [1]. In this work, we evaluate the 4D technique by comparing it against conventional cine and coronary MRA protocols.

## Methods

A Gd-BOPTA enhanced, ungated spoiled GRE sequence with 3DPR trajectory was used at 3T achieving whole-heart coverage, (1.0 mm)^3^ resolution, and fixed 10-min scan time. During offline reconstruction, data were binned into respective cardiac and respiratory phases based on motion information extracted from self-gating projections using principal component analysis, and respiratory motion was corrected using an image-based approach [2]. Then, the LV function parameters were calculated from a 16-phase 4D reconstruction, from which the quiescent period was also identified for coronary visualization. The LV end systolic volume (ESV), end diastolic volume (EDV) and ejection fraction (EF) were compared on 9 healthy subjects with a 2D multi-slice breath-hold cine protocol [3]. The quality of coronary depiction, evaluated in terms of apparent SNR/CNR (aSNR/aCNR) and vessel sharpness, were compared on 3 healthy subjects with two contrast-enhanced coronary MRA protocols with prospective ECG gating: 3DPR with respiratory motion correction [4] and Cartesian with navigator gating [5].

## Results

**Figure 2:**
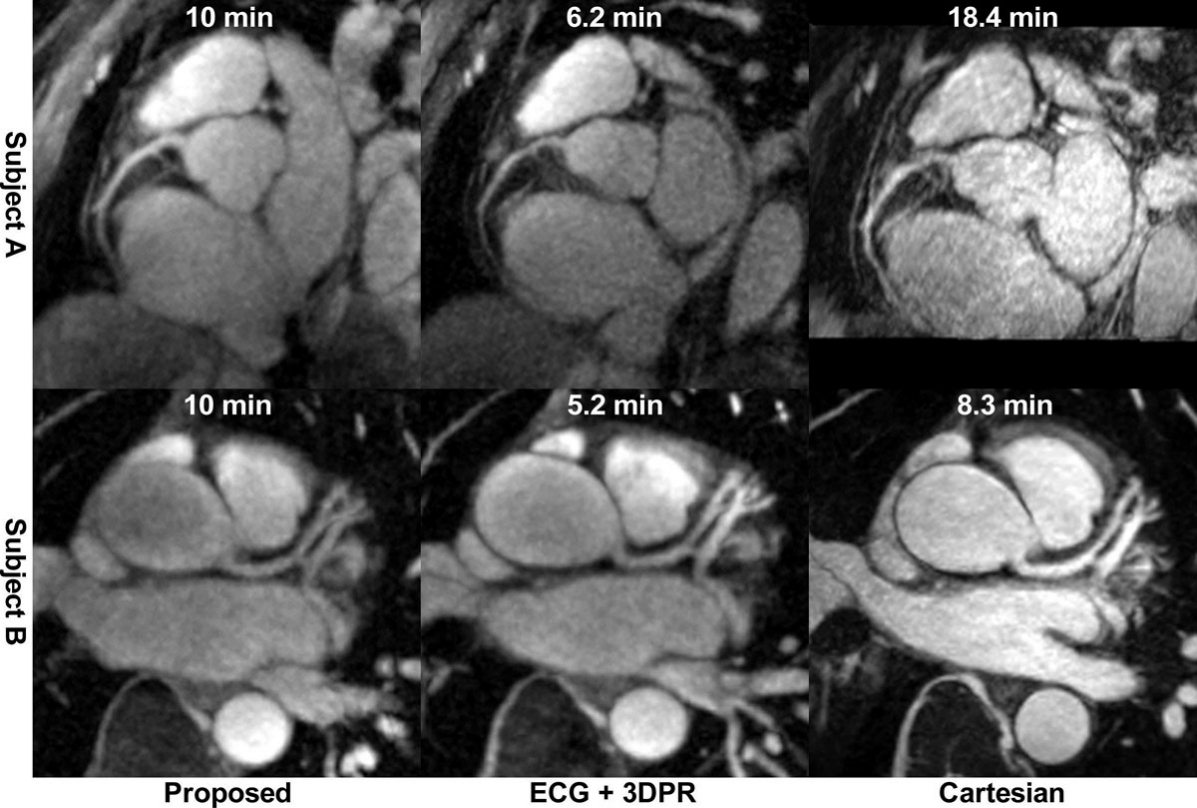
Example images from the three coronary MRA techniques of two subjects. Also shown is the scan time for each image.

Shown in Fig. 1a and 1b, the LV function parameters showed excellent correlation and agreement (one subject excluded due to poor ECG). No statistically significant differences were found (ESV: *P* = 0.10, EDV: *P* = 0.94, EF: *P* = 0.17). Shown in Fig. 1c, for the proposed, ECG+3DPR and Cartesian protocol, the mean scan times were 10.0±0.0 min (cine + coronary MRA), 6.4±1.1 min and 15.7±5.3 min (coronary MRA only), the mean sharpness were 0.35±0.08, 0.36±0.10 and 0.41±0.06 mm^-1^, the mean aSNR were 12.4±3.8, 12.8±1.1 and 12.9±2.5, and the mean aCNR were 4.5±1.5, 6.8±0.3, and 10.2±3.0, respectively. Example images are shown in Fig. 2 for two subjects.

**Figue 1:**
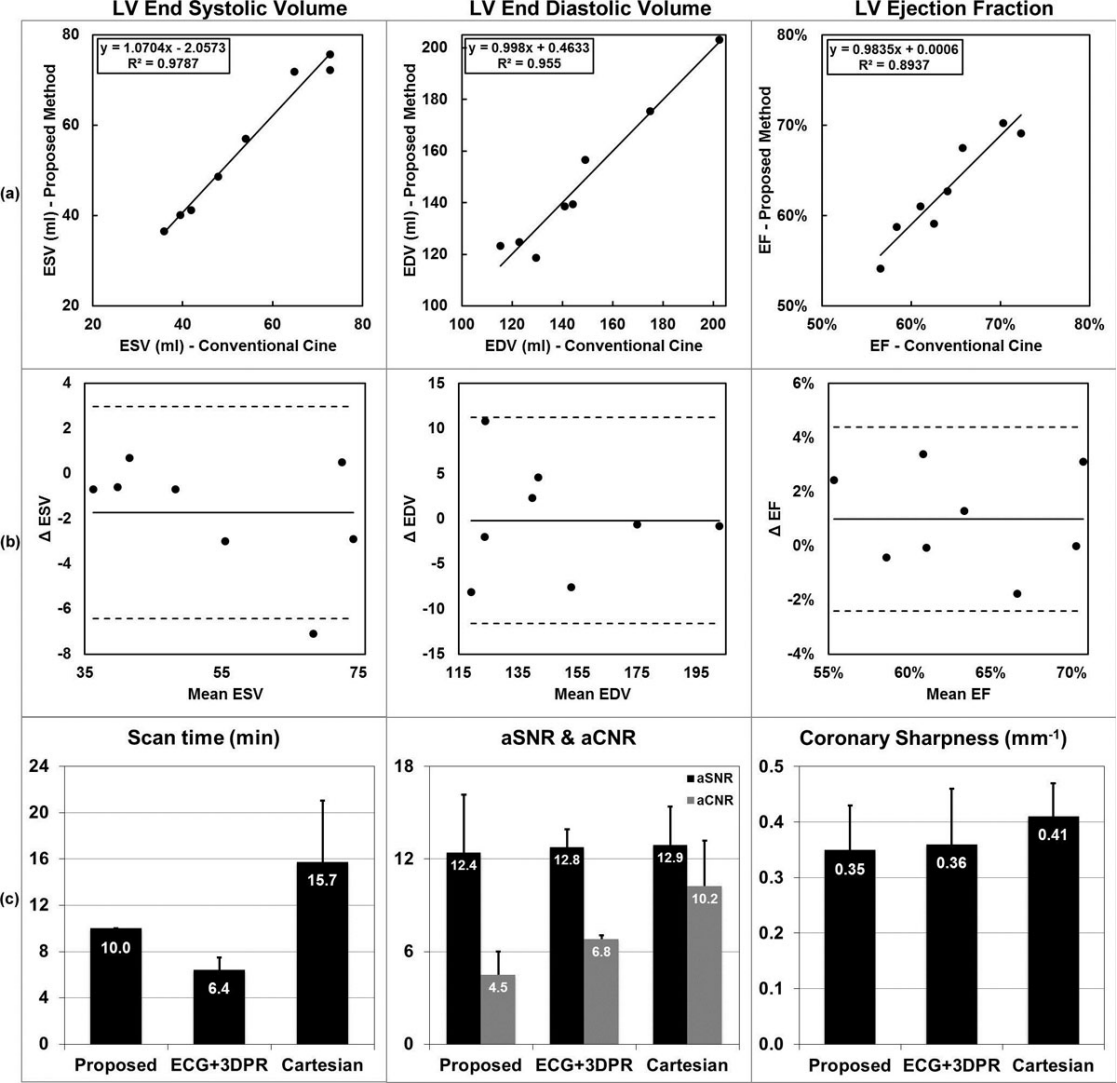
For the LV ESV, EDV and EF measurements, good correlation and agreement were found between the 4D and conventional 2D techniques, as shown in the regression (a) and Bland-Altman analysis (b). No significant differences were found between the two. Comparing with the ECG-gated coronary MRA protocols (c), the proposed method offered a fixed 10-min scan time which also included cine. The scan time of ECG+3DPR depended on the subject's heart rate, and the scan time of Cartesian depended on the subject's breathing pattern as well; the three techniques provided similar aSNR; the aCNR of the proposed method, which depended on the steady-state T1 weighting, was lower than those of the other two IR-prepared techniques; the coronary sharpness was comparable for the three techniques.

## Conclusions

In this preliminary validation, the 4D technique yielded LV function parameters in agreement with the conventional 2D cine protocol, and comparable aSNR and coronary sharpness, and lower aCNR compared with conventional ECG-gated coronary MRA protocols. Future efforts will be focused on more systematic validation on both healthy and CAD patient population, and further optimization of the 4D acquisition and reconstruction framework.

## Funding

NIH grant numbers: HL38698 and EB002623 (DL)

American Heart Association Scientist Development Grant grant number:

14SDG20480123 (BS).
